# Liquid biopsy-based comprehensive gene mutation profiling for gynecological cancer using CAncer Personalized Profiling by deep Sequencing

**DOI:** 10.1038/s41598-019-47030-w

**Published:** 2019-07-18

**Authors:** Naoyuki Iwahashi, Kazuko Sakai, Tomoko Noguchi, Tamaki Yahata, Hitomi Matsukawa, Saori Toujima, Kazuto Nishio, Kazuhiko Ino

**Affiliations:** 1 Department of Obstetrics and Gynecology, Wakayama Medical University, 811-1 Kimiidera, Wakayama, Wakayama 641-0012 Japan; 2 Department of Genome Biology, Kindai University Faculty of Medicine, 377-2 Ohno-higashi, Osaka-Sayama, Osaka 589-8511 Japan

**Keywords:** Endometrial cancer, Ovarian cancer, Cervical cancer

## Abstract

Liquid biopsies of circulating tumor DNA (ctDNA) have recently been used as a non-invasive diagnostic tool for detecting tumor-specific mutations. We present a study of ctDNA liquid biopsies in gynecological cancer using an ultrasensitive next-generation sequencing-based method for ctDNA detection named CAncer Personalized Profiling by deep Sequencing (CAPP-Seq). We performed CAPP-Seq with plasma-ctDNA obtained from 16 patients with gynecological cancer. In all cases, at least one non-synonymous somatic mutation was detected in the ctDNA. In the pre-treatment ctDNA, 4 of 16, 4/16, 5/16, 2/16, 2/16, and 2/16 patients had *TP53*, *KRAS*, *APC*, *PIK3CA*, *BRCA1*, and *EGFR* mutations, respectively. *MET* gene copy-number gains were detected in the ctDNA of 2 of 16 patients, and FISH analysis of the paired tumor samples confirmed these results. In 2 neoadjuvant chemotherapy-treated ovarian cancer patients, the changes in gene mutation patterns were associated with the treatment response. These findings suggest that CAPP-Seq-based liquid biopsies can be used for the genetic characterization of independent gynecological cancers with high frequency, and might be clinically useful for non-invasive tumor genotyping and therapeutic response monitoring.

## Introduction

Recent studies have shown that genomic alterations in solid cancers can be characterized by sequencing circulating tumor DNA (ctDNA)^1–3^. This technique is effectively a form of “liquid biopsy”, and such examinations are more widely available and easier to process than standard tumor biopsies^4,5^. DNA fragments are released into the bloodstream from apoptotic or necrotic cells^6^. In patients with solid tumors, ctDNA is also released via necrosis, autophagy, apoptosis, and other physiological events induced by microenvironmental stress as well as the effects of treatment^7^. Recent improvements in polymerase chain reaction (PCR)-based assays for analyzing blood samples for ctDNA have provided rapid, cost-effective, and non-invasive alternatives to tumor biopsies. These methods provide information about molecular alterations due to point mutations, including tumor-specific mutations, and have been used as diagnostic, prognostic, and therapeutic decision-making tools, especially in lung cancer (to detect epidermal growth factor receptor [*EGFR*] and Kirsten rat sarcoma viral oncogene homolog [*KRAS*] mutations) and colorectal cancer (to detect RAS mutations)^8–10^. Furthermore, the analysis of ctDNA liquid biopsy samples, whose results might not be influenced by heterogeneity, could aid the design of effective treatment strategies^11^. However, most early-advanced stage solid tumors exhibit very low levels of ctDNA, which makes difficult to detect genomic alterations. Although extensive research into the genetic profiling of gynecological cancers has been conducted using tumor tissue-derived DNA sequencing^12–14^, the ctDNA liquid biopsy technique has not been commonly used in the gynecological oncology field.

To improve ctDNA detection, an ultrasensitive next-generation sequencing-based (NGS-based) method for ctDNA detection named CAncer Personalized Profiling by deep Sequencing (CAPP-Seq) was recently established^15,16^. CAPP-Seq is the first NGS-based ctDNA analysis method that achieves both an ultralow detection limit and broad patient coverage at a reasonable cost and low ctDNA input level, and hence, it allows the quantitation of ctDNA from early stage tumors. CAPP-Seq achieves similar sensitivity at hotspot alleles as digital PCR or amplicon-based approaches, but is able to simultaneously interrogate thousands of additional genomic positions without its sensitivity or specificity being affected. This ultrasensitive technique can detect ctDNA in patients with early and advanced stages of various human malignancies, including lung cancer, lymphoma, and leiomyosarcoma^16–18^. In the gynecological oncology field, there have been few reports about the comprehensive gene mutation analysis of liquid biopsy samples^19–21^. Thus, we first conducted a study of multiple gene alteration analysis of ctDNA using CAPP-Seq in patients with gynecological cancer, and compared the findings with those obtained using tumor DNA sequencing.

## Results

### Clinical characteristics of the study cohort

Of the 16 patients included in this study, 2 had metastases from colorectal cancer to the ovary (COL), 4 had primary ovarian cancer (OVA), 4 had cervical cancer (CER), 5 had endometrial cancer (END), and one had uterine leiomyosarcoma (LMS). Information regarding tumor stage, treatment, and histopathological features are summarized in Table 1. Of the 16 patients, 6, 2, 4, and 3 patients were diagnosed with stage I, stage II, stage III, and stage IV disease, respectively. Pre-treatment plasma samples were available for 14 of the 16 patients who underwent surgical resection or CCRT (50 Gy radiotherapy combined with 6 cycles of weekly 40 mg/m^2^ cisplatin). Paired pre-neoadjuvant chemotherapy (NAC) and post-NAC (pre-surgery) plasma samples were available for 2 of the 16 patients who underwent interval debulking surgery after NAC. Three cycles of paclitaxel (175 mg/m^2^) and carboplatin (area under the curve: 5.0, according to Calvert’s formula) were administered every 3–4 weeks as NAC.

**Table 1:** Clinical and pathological characteristics of the study cohort.

Tumor type	Sample ID	Stage	Treatment	Histopathology
Metastatic colorectal cancer to the ovary	COL1	IV	surgery	Metastatic adenocarcinoma
	COL2	IV	surgery	Metastatic adenocarcinoma
Ovarian cancer	OVA1	IV	surgery	High grade serous carcinoma
	OVA2	III	NAC + surgery	High grade serous carcinoma
	OVA3	III	NAC + surgery	Mucinous carcinoma
	OVA4	I	surgery	Endometrioid carcinoma G1
Cervical cancer	CER1	III	CCRT	Squamous cell carcinoma
	CER2	II	CCRT	Neuroendocrine carcinoma
	CER3	II	CCRT	Squamous cell carcinoma
	CER4	I	surgery	Adenosquamous carcinoma
Endometrial cancer	END1	III	surgery	Endometrioid carcinoma G1
	END2	I	surgery	Endometrioid carcinoma G3
	END3	I	surgery	Endometrioid carcinoma G2
	END4	I	surgery	Endometrioid carcinoma G1
	END5	I	surgery	Endometrioid carcinoma G1
Uterine leiomyosarcoma	LMS1	II	surgery	Leiomyosarcoma

Abbreviations: NAC; neoadjuvant chemotherapy, CCRT; concurrent chemoradiotherapy.

### Performance of CAPP-Seq of ctDNA from patients with gynecological cancer

**Figure 1:**
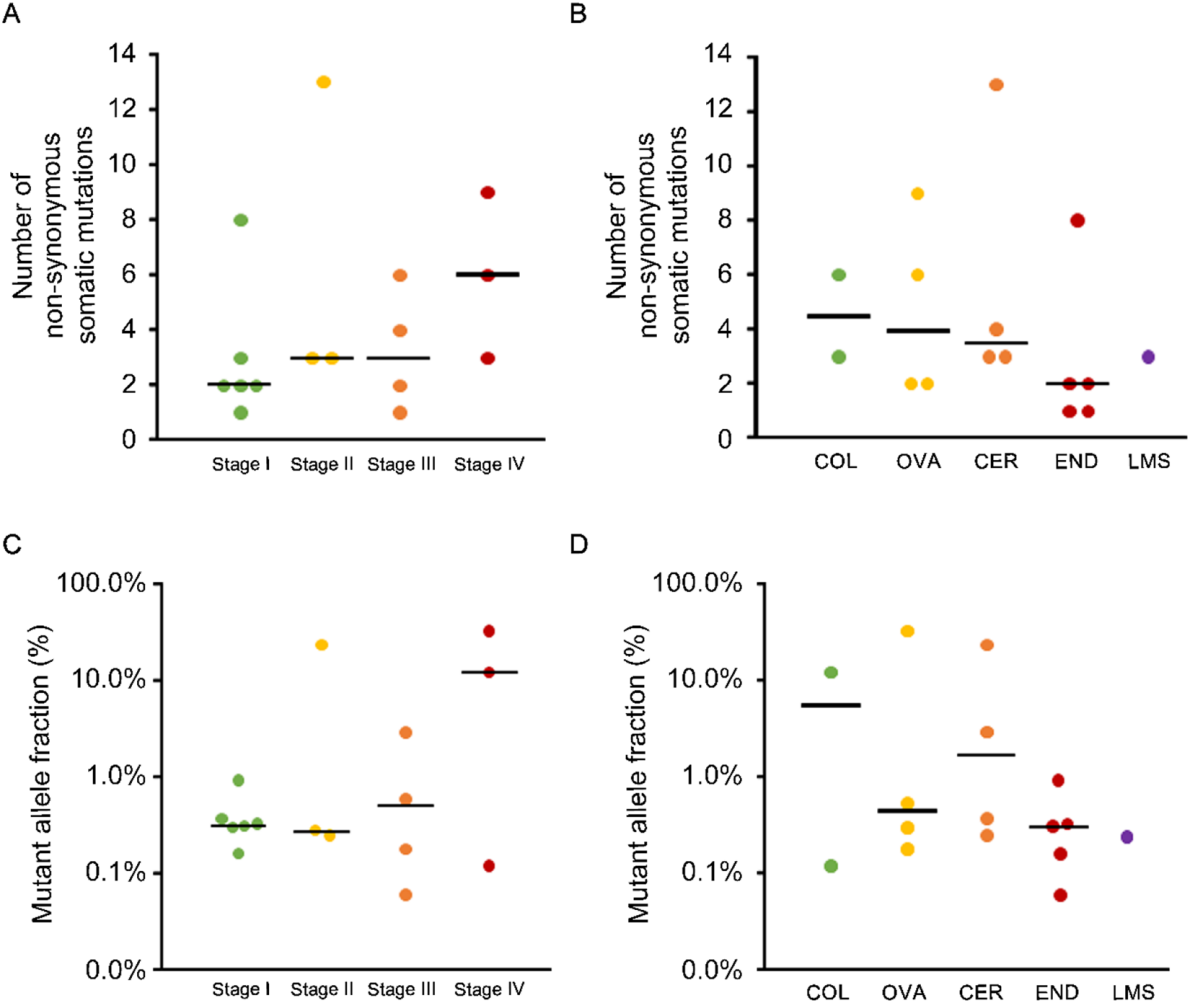
CAPP-Seq-based ctDNA analysis in patients with gynecological cancer. (**A**) The numbers of non-synonymous somatic mutations detected in the ctDNA of cancer patients according to disease stage. (**B**) The numbers of non-synonymous somatic mutations detected in the ctDNA of cancer patients according to cancer type. (**C**) Mutant allele fractions of ctDNA according to disease stage. (**D**) Mutant allele fractions of ctDNA according to cancer type. The median values for each group are represented by black bars. For patients in whom multiple alterations were detected, the highest value is shown.

The ctDNA extracted from the pre-treatment plasma samples was subjected to CAPP-Seq using a gene-sequencing panel containing 197 target genes. At least one non-synonymous somatic mutation (median: 3, range: 1–13 mutations/sample) was detected in the samples from each of the 16 patients (16/16; 100%). The number of non-synonymous somatic mutations tended to be higher in the stage IV group, although the difference was not significant (Fig. 1A). However, there were highly mutated cases in both the advanced stage and early stage groups (Fig. 1A). The number of mutations varied among the different types of cancer, with endometrial cancer having the lowest number of mutations (Fig. 1B). The highest mutant allele fraction tended to be greater in the stage IV group, although the differences between the stage IV group and the other groups were not statistically significant (Fig. 1C). There were no significant differences in the highest mutant allele fraction among the various cancer types (Fig. 1D).

**Figure 2:**
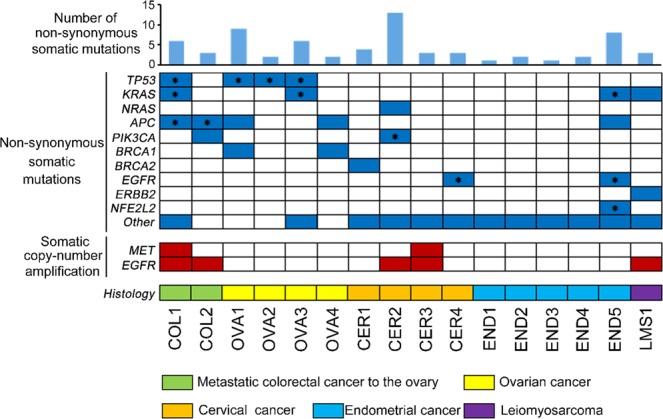
Summary of ctDNA gene alterations identified by CAPP-Seq. *Gene mutations identified in both tumor and plasma samples.

We identified 68 non-synonymous somatic mutations across 44 genes in the pre-treatment plasma ctDNA of the 16 patients (Fig. 2). In total, 4 (25%), 4 (25%), 5 (31%), 2 (13%), 2 (13%), and 2 (13%) patients had tumor protein 53 (*TP53*), *KRAS*, adenomatous polyposis coli (*APC*), phosphatidylinositol-4,5-bisphosphate 3-kinase, catalytic subunit alpha (*PIK3CA*), breast cancer gene 1 (*BRCA1*), and *EGFR* mutations, respectively. Of the 68 non-synonymous somatic mutations detected in the plasma ctDNA, 13 were also observed in the paired tumor DNA (analyzed by QIAseq using a gene sequencing panel containing 275 target genes). Concordance between the mutations detected in the plasma and tumor DNA was evident in four cancer types; i.e., in 2 of 2 (100%) patients with metastasis from colorectal cancer to the ovary, 3 of 4 (75%) patients with ovarian cancer, 2 of 4 (50%) patients with cervical cancer, and 1 of 5 (20%) patients with endometrial cancer. The plasma/tumor-matched mutations included 4 *TP53* mutations (H193Y, Y205C, Y234N, and R248W), 2 *KRAS* mutations (G12D and G13D), 2 *APC* mutations (R216* and E1306*), one *PIK3CA* mutation (E545K), 2 *EGFR* mutations (A611T and R831H), and one nuclear factor (erythroid-derived 2)-like 2 (*NFE2L2*) mutation (R34G).

### Copy-number amplification in the ctDNA extracted from the plasma samples

**Figure 3:**
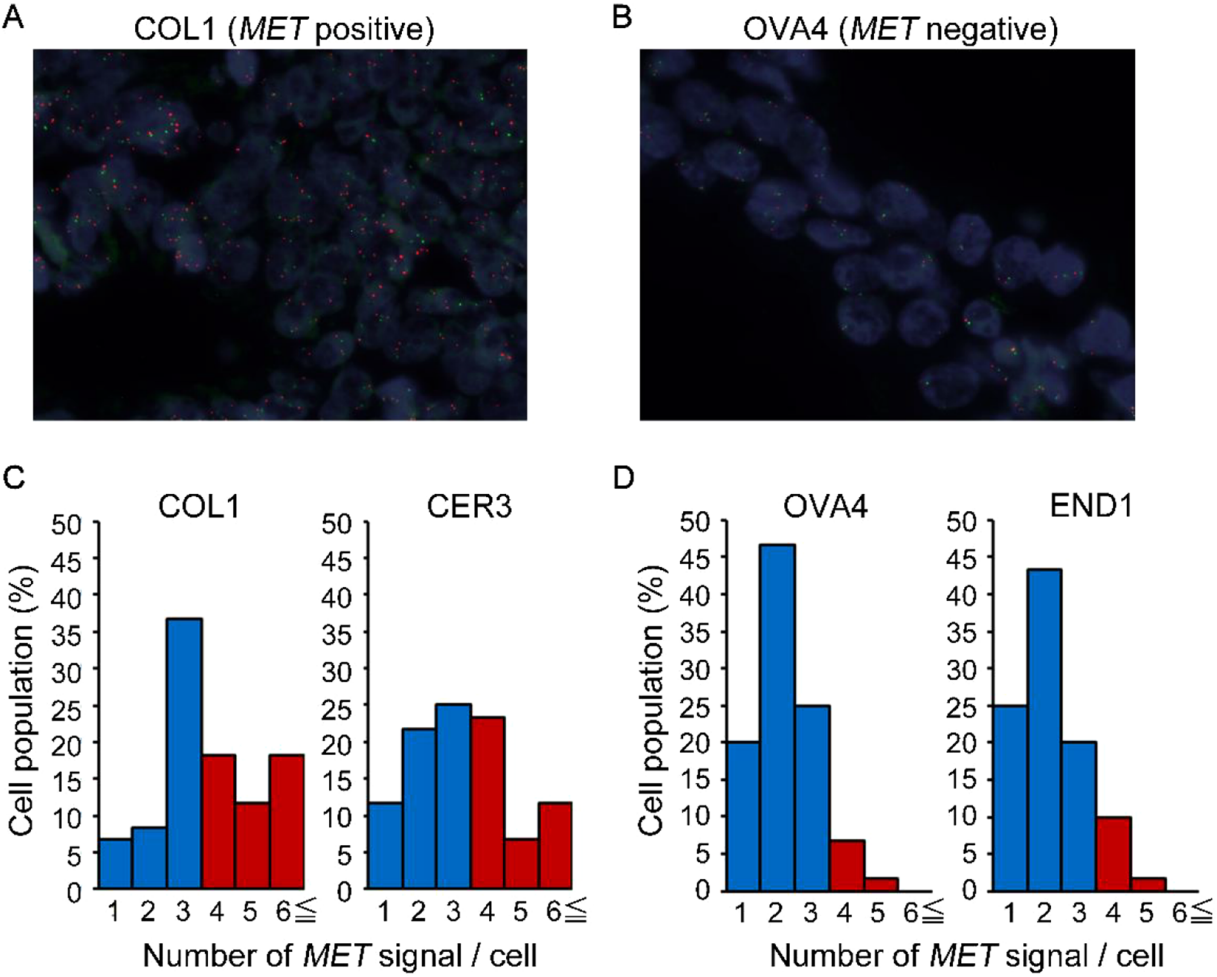
FISH analysis of the MET gene. (**A**) Image of a FISH-positive specimen (COL1). (**B**) Image of a FISH-negative specimen (OVA4). (**C**) Number of MET signals/cell in FISH-positive specimens (COL1 and CER3). (**D**) Number of MET signals/cell in FISH-negative specimens (OVA4 and END1).

We also detected somatic copy-number amplification in two genes, *MET* and *EGFR*, in the plasma ctDNA using CAPP-Seq (Fig. 2). *MET* copy-number amplifications were observed in 2 patients (13%), and *EGFR* copy-number amplifications were observed in 5 patients (31%). To investigate the validity of the *MET* copy-number amplification detected by CAPP-Seq, the paired tumor FFPE specimens were subjected to FISH analysis of *MET* amplification (Fig. 3). In the 2 patients that displayed *MET* amplification during CAPP-Seq (COL1 and CER3), 48.3% (Fig. 3A,C) and 41.7% (Fig. 3C) of the FFPE specimen cells contained ≥4 copies of the *MET* gene, respectively. While in 2 patients that did not demonstrate *MET* amplification during CAPP-Seq (OVA4 and END1), 8.4% of FFPE specimen cells contained ≥4 copies of the *MET* gene in OVA4 (Fig. 3B,D), and 11.7% of FFPE specimen cells were in END1 (Fig. 3D).

### Plasma ctDNA monitoring for assessing the response to NAC

**Figure 4:**
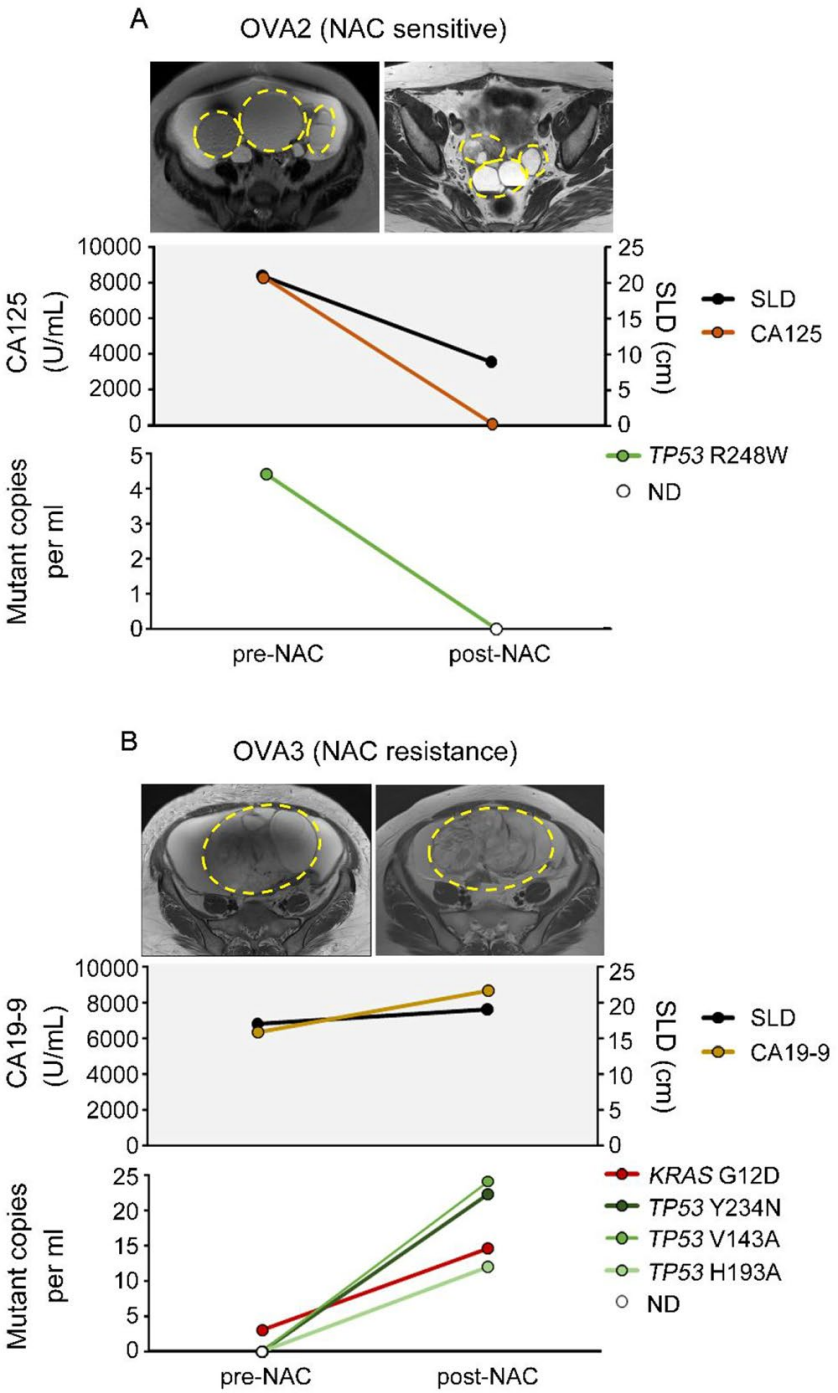
Changes in gene mutation profiles of ctDNA and clinical courses in NAC-treated ovarian cancer cases. Comparison of gene mutations in pre- and post-NAC ctDNA and the clinical courses of (**A**) an NAC-sensitive ovarian cancer case (OVA2) and (**B**) an NAC-resistance ovarian cancer case (OVA3).

Our results demonstrated the possibility of using CAPP-Seq for ctDNA to assess treatment responses in ovarian cancer patients treated with NAC (Fig. 4). In 2 ovarian cancer patients, OVA2 and OVA3, ctDNA was isolated from both their pre-NAC and post-NAC plasma samples, and CAPP-Seq was performed to monitor the changes in the gene mutation patterns seen after NAC. In the NAC-sensitive case OVA2 (Fig. 4A), the sum of the longest diameters (SLD) of the tumor decreased from 21 to 9 cm, and the cancer antigen 125 (CA125) level decreased from 8275 to 52.9 U/mL. The maximum standardized uptake value (SUVmax) on 18F-fluoro-2-deoxy-D-glucose positron emission tomography combined with computed tomography (FDG-PET/CT), which is a parameter of tumor aggressiveness, also decreased from 12.4 to 2.96. We detected one *TP53* mutation (R248W; mutation level: 4.42 mutant copies per mL) in the patient’s pre-NAC ctDNA via CAPP-Seq, while no *TP53* (R248W) mutations were detected in their post-NAC ctDNA. In the analysis of the post-NAC FFPE tissue sample from OVA2, we detected the same *TP53* mutation (R248W) as was detected in the patient’s pre-NAC ctDNA. In the NAC-resistant case OVA3 (Fig. 4B), the SLD of the tumor increased from 17 to 19 cm, and the patient’s CA19-9 level increased from 6342 to 8690 U/mL. We detected one *KRAS* mutation (G12D; mutation level: 3.04 mutant copies per mL) in the patient’s pre-NAC ctDNA via CAPP-Seq, while their post-NAC ctDNA exhibited an increased *KRAS* (G12D) mutation level (14.6 mutant copies per mL) and 3 novel *TP53* mutations (Y234N, V143A, and H193A). In the analysis of the post-NAC FFPE tissue sample from OVA3, we detected the same *KRAS* (G12D) mutation and one of the *TP53* (Y234N) mutations that were detected in the patient’s post-NAC ctDNA.

## Discussion

To the best of our knowledge, the current study is the first to demonstrate the utility of analyzing comprehensive gene alterations of liquid biopsy-derived ctDNA using CAPP-Seq in patients with gynecological cancer. Using CAPP-Seq, we detected at least one non-synonymous somatic mutation in the plasma ctDNA of all 16 gynecological cancer patients, and 8 of 16 patients had mutations in their plasma that matched mutations found in their paired tumor samples. These findings suggest that CAPP-Seq-based ctDNA profiling of gynecological malignancies is useful.

The use of targeted hybrid capture with high-throughput sequencing and a specialized bioinformatics workflow technique for plasma ctDNA allows the highly sensitive, non-invasive, and low-cost ctDNA-detection^15,16^. CAPP-Seq has been demonstrated to be clinically useful for improving prognostication in lymphoma^17^ and for studying the heterogeneity of resistance mechanisms in lung cancer patients^22^. Plasma genotyping of *EGFR*-sensitizing mutations has already been approved by the US Food and Drug Administration as a technique for selecting feasible drugs^10,23^. In our study, we determined the gene mutation profile of ctDNA using CAPP-Seq, not only in gynecological cancer, but also in metastasis from colorectal cancer to the ovary, which exhibited the well-known genetic signature of colorectal cancer (*KRAS*, *APC*, and *TP53* mutations and a *MET* copy-number gain). Therefore, CAPP-Seq might be applicable to a broad range of clinical cases and have the potential to accelerate the personalized detection, treatment, and monitoring of many kinds of cancer.

In the gynecological oncology field, there have only been a few reports about gene mutation analysis using ctDNA. We revealed that CAPP-Seq-based examinations have the potential to facilitate the characterization of the genetic profiles of gynecological cancer. All 16 of the analyzed plasma samples had at least one detectable non-synonymous somatic mutation according to the ultrasensitive NGS-based method employed in this study. FFPE tumor samples were also examined, and the same mutation was detected in both the tumor and plasma samples in 8 patients. These results suggest that CAPP-Seq is feasible as an ultrasensitive method for performing comprehensive gene mutation analysis of gynecological cancer, as has been reported for lung cancer, lymphoma, and leiomyosarcoma^16–18^. Chabon *et al*. used CAPP-Seq to examine the intra-patient tumor heterogeneity that arose during treatment for non-small cell lung cancer^22^. Among 41 patients who exhibited T790M mutations at enrollment after receiving *EGFR*-targeted therapy, additional putative resistance mutations were detected in the pretreatment plasma ctDNA of 19 patients (46%), including *MET* copy-number gains, the target gene alteration for the *MET* inhibitor crizotinib, which we also detected in 2 patients using both CAPP-Seq of ctDNA and FISH analysis of tumor samples (COL1 and CER3). Przybyl *et al*. reported that ctDNA monitoring using CAPP-Seq was clinically useful in 7 cases of leiomyosarcoma, including 3 cases of uterine leiomyosarcoma^18^. In the latter study, gene mutations were detected in the ctDNA of 6 of 7 leiomyosarcoma patients using CAPP-Seq, and it was suggested that CAPP-Seq has potential as a method for assessing tumor heterogeneity. In our case LMS1, although we could not detect any gene mutations in 3 FFPE tumor specimens, we detected non-synonymous somatic mutations in the *KRAS* and receptor tyrosine-protein kinase erbB-2 (*ERBB2*) genes, both of which are known to be important actionable genes, in the analysis of the patient’s ctDNA. Further studies are needed to confirm the usefulness of CAPP-Seq for assessing tumor heterogeneity in gynecological cancer.

Ovarian cancer is often diagnosed at an advanced stage, and little progress has been made in the existing chemotherapies for the disease^24^. Although there are several new therapeutic concepts that can be used to treat some cases of ovarian cancer, including targeted therapy involving the vascular endothelial growth factor inhibitor bevacizumab or the poly-(ADP-ribose) polymerase inhibitor olaparib^25,26^, ovarian cancer still causes the majority of cancer-related deaths from gynecological cancer^27^. Thus, a new treatment strategy for ovarian cancer, including novel approaches for early diagnosis, tumor monitoring, and/or detecting novel molecular targets, is needed. There have only been a few studies about the comprehensive gene mutation analysis of ctDNA in the gynecological oncology field, and they involved small numbers of patients^19–21^. Phallen *et al*.^19^ used targeted error correction sequencing, which allows direct ultrasensitive evaluations of 58 cancer-related genetic alterations, to analyze ctDNA from four types of cancer (colorectal, lung, ovarian, and breast cancer). In their study, of the 42 ovarian cancer patients included, 27 (64%) had at least one detectable somatic mutation in their plasma ctDNA (range: 1–3 mutations/sample), and about 50% of the ovarian cancer patients had *TP53* mutations. Cohen *et al*.^20^ used CancerSEEK, a combined assay for genetic alterations in 16 cancer-related genes and 41 protein biomarkers, to analyze eight types of cancer (colorectal, lung, ovarian, breast, liver, stomach, pancreatic, and esophageal cancer). Although the 54 ovarian cancer patients included in the latter study had detectable somatic mutations in their plasma ctDNA, only one somatic mutation was detected in each patient, and 46 (85%) patients had *TP53* mutations. Arend *et al*.^21^ used a basic NGS method (the Ion AmpliSeq cancer hotspot panel v2) for ultra-deep targeted sequencing of ctDNA from 14 patients with advanced ovarian cancer (stage III–IV). Although they detected 12 variants in matched tumor and plasma ctDNA samples, it was considered likely that all 12 of these variants were germline mutations. In our study, all of the ovarian cancer patients (4/4; 100%) had at least one detectable non-synonymous somatic mutation, and 3 of the 4 patients had identical non-synonymous somatic mutations in their matched tumor and plasma ctDNA samples. Indeed, our results demonstrate the possibility of performing ctDNA liquid biopsy examinations using CAPP-Seq for monitoring treatment responses, including “tumor evolution^28^”; i.e., evolutionary changes in tumor heterogeneity, in NAC-treated patients with advanced ovarian cancer (OVA2 and OVA3). In our NAC-sensitive case OVA2 (Fig. 4A), CAPP-Seq detected a reduction in the *TP53* (R248W) mutation level after NAC treatment. In contrast, in the NAC-resistant case OVA3 (Fig. 4B), we detected an increase in the *KRAS* (G12D) mutation level and the appearance of 3 novel *TP53* mutations (Y234N, V143A, and H193A) after NAC treatment. Thus, CAPP-Seq might be a useful tool for non-invasive tumor genotyping as well as for therapeutic response monitoring, detecting evolutional changes in tumor heterogeneity.

The information about ctDNA liquid biopsies of uterine cancer (cervical and endometrial cancers) is even more limited than that about ovarian cancer^29,30^. There have not been any reports about the comprehensive gene mutation analysis of ctDNA in uterine cancer, and the current study is the first to characterize the ctDNA genetic profile of uterine cancer by CAPP-Seq. Chung *et al*. assessed the frequency of 2 major *PIK3CA* mutations in uterine cervical cancer, E542K and E545K, using droplet digital PCR of plasma ctDNA, and the detection of *PIK3CA* mutations in plasma was associated with reduced disease-free survival and overall survival^29^. Bolivar *et al*. used a custom-designed Accel-Amplicon NGS panel covering hotspots in the phosphatase and tensin homolog (*PTEN*), catenin beta-1 (*CTNNB1*), *KRAS*, and *PIK3CA* genes to analyze ctDNA from 48 endometrial cancer patients^30^. At least one mutation was detected in the ctDNA of 17 (35%) patients, and the same mutation was detected in both tumor and plasma samples in 15 of 45 patients (33%), which demonstrated that mutations in endometrial cancer-related genes can be identified in plasma ctDNA. We detected at least one non-synonymous somatic mutation in all of the uterine cancer patients in this study (CER and END), and identical non-synonymous somatic mutations were detected in matched tumor and plasma ctDNA samples in 3 of the 9 patients (CER2, CER4, and END5). Indeed, we also detected a *MET* copy-number gain in one patient (CER3) and *EGFR* copy-number gains in 2 patients (CER2 and CER3), both of which could be targets for novel molecular treatments for uterine cancer.

Interestingly, our study showed that there might be populations of highly mutated tumors in both ovarian and uterine cancer, which could be detected by CAPP-Seq (e.g., OVA1, CER2, and END5). Rizvi *et al*. reported that the tumor mutation burden (TMB), as determined by targeted NGS, might be associated with the response to immunotherapy in patients with lung cancer^31^. Gandara *et al*. demonstrated that the TMB according to blood tests (blood-TMB; bTMB) might be associated with the clinical benefit from immune checkpoint inhibitor therapy in patients with lung cancer^32^. In addition, it was reported that CAPP-Seq has potential as a technique for measuring the bTMB among early-advanced stage cancer and for monitoring ctDNA during treatment^15,16^. Further study is necessary to determine the utility of performing ctDNA liquid biopsies using CAPP-Seq for evaluating the bTMB in the gynecological field.

Collectively, the present study has demonstrated the utility of performing ctDNA liquid biopsy examinations, which are non-invasive and easy to perform repeatedly, using the novel ultrasensitive CAPP-Seq method in patients with gynecological cancer. Genetic characterization of gynecological cancers based on CAPP-Seq might aid the development of novel personalized treatment strategies. There are some limitations to our study. We did not utilize the tumor DNA for CAPP-Seq in the same gene-sequencing panel. CAPP-Seq is currently specialized for plasma ctDNA, and it therefore remains a future challenge to expand its application to formalin-fixed paraffin-embedded tumor tissue. Further experiments using the tumor DNA are needed to explore the ability/sensitivity to detect the tumor mutations by ctDNA. Another limitation is that we conducted the retrospective study cohort with a relatively small number of patients. Further studies should be in a large number of patients conducted to clarify the clinical usefulness of CAPP-Seq-based ctDNA profiling.

## Material and Methods

### Ethical approval and informed consent

The present study was approved by the ethics committees of Wakayama Medical University (Authorization Number: 2025) and Kindai University Faculty of Medicine (Authorization Number: 29-066). All of the patients in this study provided written informed consent for the use of their plasma and tissue samples. All methods were performed in accordance with the Declaration of Helsinki specified by Wakayama Medical University and Kindai University Faculty of Medicine.

### Patients and samples

A total of 16 patients with gynecological cancer who underwent surgical resection, chemotherapy, or concurrent chemoradiotherapy (CCRT) at Wakayama Medical University Hospital between May 2017 and January 2018 were included in this study. The tumor staging was carried out according to the International Federation of Gynecology and Obstetrics (FIGO) classification, and tumor grading was performed according to the World Health Organization (WHO) classification. All patients were pathologically diagnosed using surgical resection or biopsy samples. Histological diagnosis was determined on the basis of standard H&E-stained sections by two or more experienced senior pathologists.

### Circulating tumor DNA extraction and sequencing

CAPP-Seq was performed with ctDNA isolated from plasma samples as previously reported^16^. In brief, we used a maximum of 50 ng of DNA for the CAPP-Seq ctDNA analyses, which were performed using the AVENIO ctDNA surveillance kit for 197 genes (Roche Diagnostics, Indianapolis, IN), according to the manufacturer’s instructions. The AVENIO ctDNA Surveillance Kit contains major mutated genes in gynecological cancer (*TP53*, *BRCA1*, *BRCA2*, *BRAF*, *PIK3CA*, *KRAS*, *NRAS*, *APC*, *EGFR*, *MET*, *ERBB2*, *CTNNB1*, etc.). The purified libraries were pooled and sequenced on an Illumina NextSeq. 500 (Illumina, San Diego, CA) using a 300-cycle high-output kit. Variants were called with the AVENIO ctDNA analysis software (Roche Diagnostics), which includes bioinformatics methods from CAPP-Seq^15^ and integrated digital error suppression^16^. Germline mutations were excluded based on the Human Genetic Variation Database (http://www.genome.med.kyoto-u.ac.jp/SnpDB) and the Exome Aggregation Consortium database^33^.

### Tumor DNA extraction and sequencing

We analyzed 40 ng of DNA with the QIAseq Human Comprehensive Cancer Panel for 275 genes (Qiagen, Valencia, CA). The library preparation was performed according to the manufacturer’s instructions^34^. The purified libraries were pooled and then sequenced with a NextSeq. 500 instrument (Illumina). Reads were aligned with the hg19 human reference genome, and variant detection was performed according to the manufacturer’s pipeline. Germline mutations were excluded using the Human Genetic Variation Database (http://www.genome.med.kyoto-u.ac.jp/SnpDB) and the Exome Aggregation Consortium database^33^.

### Fluorescence *in situ* hybridization analysis (FISH)

Amplification of the *MET* gene from tumor tissue was examined via FISH analysis. Four-micrometer FFPE specimens were hybridized with the MET (TexRed)/CEN7q (FITC) dual-color FISH probe (GSP Laboratory, LCI Medience Corporation, Chiba, Japan). The number of fluorescent signals was counted independently by two investigators. Positivity for *MET* gene amplification was defined as follows: over 30% of cells exhibiting ≥4 copies of the *MET* gene. These examinations were outsourced to LSI Medience Corporation (Tokyo, Japan).

### Statistical methods

The statistical analyses were performed using the Mann-Whitney *U* test. *P*-values of <0.05 were considered significant.

## Data Availability

The data that support the findings of this study are available from the corresponding author upon reasonable request.
